# 慢性移植物抗宿主病患者生活质量评估及其差异研究

**DOI:** 10.3760/cma.j.cn121090-20231008-00162

**Published:** 2024-01

**Authors:** 世勤 黄, 瑞昊 黄, 瑶 全, 凤鸣 王, 先静 程, 筱淇 王, 曦 张

**Affiliations:** 1 陆军军医大学第二附属医院血液病医学中心，全军临床重点专科，创伤与化学中毒国家重点实验室，重庆市医学重点学科血液病与微环境重庆市重点实验室，重庆 400037 Medical Center of Hematology, Xinqiao Hospital of Army Medical University, Chongqing Key Laboratory of Hematology and Microenvironment, State Key Laboratory of Trauma and Chemical Poisoning, Chongqing 400037, China; 2 金凤实验室，重庆 400037 Jinfeng Laboratory, Chongqing 400037, China

**Keywords:** 慢性移植物抗宿主病, 生活质量, 症状负荷, 患者报告结局, Chronic graft-versus-host disease, Quality of life, Symptom burden, Patient-Reported outcome

## Abstract

**目的:**

评估慢性移植物抗宿主病（cGVHD）患者的生活质量（QoL）现状、差异及其影响因素。

**方法:**

通过横断面研究，2021年9月至2023年2月向我中心140例cGVHD患者发放调查问卷，采用 Lee症状学量表（LSS）评估症状负荷，医学结局研究36项简短健康调查量表（SF-36）、五水平五维健康量表（EQ-5D-5L）评估生活质量。

**结果:**

在140份可评估的调查者问卷中，轻度、中度、重度cGVHD患者分别为32例（22.9％）、87例（62.1％）、21例（15.0％），男性占61.4％，中位移植年龄为34（15～68）岁。原发病类型：急性髓系白血病70例（50.0％），急性淋巴细胞白血病28例（20.0％），骨髓增生异常综合征21例（15.0％），其他21例（15.0％）。常见的cGVHD受累器官为皮肤74例（52.9％）、眼睛57例（40.7％）和肝脏50例（35.7％）。在整体患者中，LSS量表的眼睛［（20.48±23.75）分］、心理［（16.13±17.00）分］、口腔［（13.66±20.55）分］评分最高；SF-36量表的生理职能［（36.07±11.13）分］、社会功能［（36.10±10.68）分］、情感职能［（38.36±11.88）分］方面评分最低；EQ-5D效用指数值为0.764。轻度、中度、重度患者LSS量表总分分别为（6.51±6.15）、（10.07±5.61）、（20.90±10.09）分；SF-36躯体健康总评（PCS）分别为（43.12±6.38）、（40.73±7.14）、（36.97±6.97）分，精神健康总评（MCS）分别为（43.00±8.47）、（38.90±9.52）、（28.96±9.63）分；EQ-5D效用指数值分别为0.810±0.124、0.762±0.179、0.702±0.198。多因素分析结果显示，总体症状负担（*β*＝−0.517）、口腔相关症状（*β*＝−0.456）、眼睛美国国立卫生研究院（NIH）评分系统分级（*β*＝−0.376）、营养相关症状（*β*＝−0.211）与PCS呈显著负相关；眼睛NIH分级（*β*＝−0.260）与MCS呈显著负相关；口腔相关症状（*β*＝−0.400）、关节/筋膜NIH分级（*β*＝−0.332）、累及系统数量（*β*＝−0.253）、整体NIH分级（*β*＝−0.205）、服用免疫抑制剂种类（*β*＝−0.171）与EQ-5D效用指数值呈显著负相关（*P*<0.05）。EQ-5D效用指数值与SF-36呈中到高相关（|*r*|＝0.384～0.571，*P*<0.001）。

**结论:**

cGVHD患者QoL受损，疾病越重QoL越差。总体症状负荷、眼睛严重程度、口腔症状负荷是影响QoL最重要的因素。

## Abstract (en)

**Objective:**

To evaluate the status of, differences in, and factors influencing quality of life (QoL) in patients with chronic graft-versus-host disease (GVHD).

**Methods:**

From September 2021 to February 2023, a cross-sectional study of 140 patients with chronic GVHD was conducted at our center. Symptom burden was assessed by the Lee Symptomatology Scale (LSS), and QoL was assessed by the Medical Outcome Study 36-Item Short-Form Health Survey (SF-36) (version 1) and five-level EuroQoL five-dimensional questionnaire (EQ-5D-5L).

**Results:**

Data from 140 respondents, including 32 (22.9%) with mild chronic GVHD, 87 (62.1%) with moderate chronic GVHD, and 21 (15.0%) with severe chronic GVHD, were analyzed. Of the respondents, 61.4% were male, and the median transplantation age was 34 (15–68) years. The primary diagnoses were acute myeloid leukemia (50.0%), acute lymphoblastic leukemia (20.0%), and myelodysplastic syndrome (15.0%). The common chronic GVHD-affected organs included the skin in 74 patients (52.9%), the eyes in 57 patients (40.7%), and the liver in 50 patients (35.7%). Among the whole cohort, the eye (20.48±23.75), psychological (16.13±17.00), and oral (13.66±20.55) scores were highest in the LSS group. The physiological function (36.07±11.13), social function (36.10±10.68), and role-emotional functioning (38.36±11.88) scores were lowest in the SF-36 group. The EQ-5D index was 0.764. The total LSS scores for mild, moderate, and severe chronic GVHD were 6.51±6.15, 10.07±5.61, and 20.90±10.09, respectively. The SF-36 physical component scores (PCSs) were 43.12±6.38, 40.73±7.14, and 36.97±6.97, respectively, and the mental component scores (MCSs) were 43.00±8.47, 38.90±9.52, and 28.96±9.63, respectively. The EQ-5D values were 0.810±0.124, 0.762±0.179, and 0.702±0.198, respectively. The multivariate analysis showed that the overall symptom burden (*β*＝−0.517), oral symptom burden (*β*＝−0.456), National Institute of Health (NIH) criteria for the eyes (*β*＝−0.376), and nutrition-related symptom burden (*β*＝−0.211) were significantly negatively correlated with the PCS. The NIH score (*β*＝−0.260) was negatively correlated with the MCS score. Oral symptom burden (*β*＝−0.400), joint/fascia NIH criteria (*β*＝−0.332), number of involved systems (*β*＝−0.253), overall NIH criteria (*β*＝−0.205), and number of immunosuppressants taken (*β*＝−0.171) were significantly negatively correlated with the EQ-5D score (all *P*<0.05). Medium to strong correlations were found between the EQ-5D score and the SF-36 score (|*r*|＝0.384–0.571, *P*<0.001).

**Conclusion:**

The QoL of patients with chronic GVHD is impaired, and the more severe the disease, the poorer the QoL. Overall symptom burden, severity of eyes, and oral symptom burden were the most important factors affecting QoL.

异基因造血干细胞移植（allo-HSCT）是治愈血液系统恶性肿瘤的有效治疗方法[1]–[3]，慢性移植物抗宿主病（cGVHD）是移植后最常见的远期并发症及非复发死亡原因，发生率为30％～70％，病程迁延不愈，累及全身脏器[4]–[5]。疾病的复杂性和药物的不良反应严重影响患者的生活质量（QoL），如何改善和提高患者的QoL广受关注[6]–[8]。QoL是患者报告结局（PRO）的重要组成部分，包括健康和疾病的躯体、心理和社会功能状态等方面[9]–[10]。研究表明，cGVHD的发生和严重程度是allo-HSCT后QoL的最主要决定因素[11]–[13]。然而，我们在血液病患者移植后的管理中并没有QoL的标准测量工具，也缺乏中国样本的验证和推荐。为此，我们设计了一项横断面观察性研究，以评估中国cGVHD患者的QoL现状、人群差异及影响因素。

## 病例与方法

一、研究设计

以问卷调查的方式收集2021年9月至2023年2月我中心cGVHD患者的数据。入组标准：①年龄≥16岁，具有文字读写和理解能力；②接受门诊医师提供的cGVHD诊断和治疗；③存在活跃的cGVHD症状；④自愿参与本研究。排除标准：①有精神疾病史、经历严重生活事件或失眠史；②移植后发生感染、植入不良、继发肿瘤等并发症。本研究获得陆军军医大学新桥医院伦理委员会批准（批件号：2023-研第130-01）。

二、cGVHD患者的基线和疾病特征收集

我中心移植后的患者在门诊定期随访，登记基础疾病和移植的相关信息，临床医师规范开展cGVHD的诊断和治疗，采用基于国内共识[14]即美国国立卫生研究院（NIH）评分系统[15]对受累器官依次进行评估并明确严重程度分度[16]。

三、cGVHD的症状评估工具

Lee症状学量表（LSS）：NIH工作组推荐的cGVHD特异性测量工具，包含30个症状条目，得分线性转化为0～100分，汇总为7个子量表（皮肤、眼睛、口腔、呼吸、营养、精力、心理）和1个总量表得分，分数越高代表症状负担越重[17]。

四、cGVHD的QoL评估工具

1. 医学结局研究36项简短健康调查量表（版本1）（SF-36）：非特异性cGVHD的辅助测量工具，包含36个条目，概括为8个健康概念：生理功能（PF）、生理职能（RP）、躯体疼痛（BP）、总体健康（GH）、活力（VT）、社会功能（SF）、情感职能（RE）、精神健康（MH），概括为躯体健康总评（PCS）和精神健康总评（MCS）[18]。8个子量表和2个概括量表（PCS、MCS）采用美国标准化算法和常模[19]，分数越低代表QoL越差。

2. 五水平五维健康量表（EQ-5D-5L）：描述健康状况的通用量表，包含5个条目：活动能力（MO）、自我护理（SC）、日常活动（UA）、疼痛/不适（PD）和焦虑/抑郁（AD），依次在5个层次上进行描述[20]。问卷结果由效用指数值表示，采用中国人口的效用指数值集[21]，值越低代表QoL越差。

五、统计学处理

采用标准算法计算量表得分，定量资料符合正态分布者用均值±标准差表示，计数资料以频数（构成比）表示。单因素方差分析比较不同严重程度患者间的结果差异，采用邦弗伦尼（方差齐性）或塔姆黑尼T2（方差不齐）进行事后两两比较；单因素分析*P*≤0.2的变量代入多元线性回归模型进行多因素分析；皮尔逊相关性检验分析EQ-5D效用指数值与SF-36评分的相关性。双侧*P*<0.05为差异有统计学意义。采用SPSS 25.0和Graphpad Prism 10.0软件进行数据分析和作图。

## 结果

一、受访者特征

1. 基线特征：共对140例受访者进行评估，其中男86例（61.4％），女54例（38.6％），移植时中位年龄为34（15～68）岁；原发病类型：急性髓系白血病（AML）70例（50.0％），急性淋巴细胞白血病28例（20.0％），骨髓增生异常综合征21例（15.0％），其他21例（15.0％）；59.3％的患者干细胞来源为外周血，55.7％的患者为HLA部分相合，女供男占16.4％。移植后cGVHD的中位发生时间为6.1（3.1～108.6）个月，其中53例（37.9％）移植后5个月内发生cGVHD，87例（62.1％）移植后5个月后发生cGVHD；进行量表的中位评估时间为移植后9.7（3.3～124.4）个月、cGVHD发病后1.8（0～38.5）个月。

2. cGVHD相关特征：根据NIH标准进行cGVHD分度，其中轻度32例（22.9％）、中度87例（62.1％）、重度21例（15.0％）；常见的受累器官为皮肤74例（52.9％）、眼睛57例（40.7％）和肝脏50例（35.7％），61.4％的患者累及2个及以上的系统，30.0％的患者既往发生急性GVHD（aGVHD），13.6％的患者为重叠综合征。在接受问卷评估时，37.1％的患者接受二线治疗，30.7％的患者使用2种及以上免疫抑制剂（表1）。

**表1:** 140例异基因造血干细胞移植发生慢性移植物抗宿主病（cGVHD）受访者的cGVHD特征［例（％）］

特征	结果	特征	结果
NIH分度		肺评分	
轻度	32（22.9）	0	124（88.6）
中度	87（62.1）	1	9（6.4）
重度	21（15.0）	2	7（5.0）
皮肤评分		3	0
0	66（47.1）	关节/筋膜评分	
1	53（37.9）	0	118（84.3）
2	13（9.3）	1	17（12.1）
3	8（5.7）	2	4（2.9）
口腔评分		3	1（0.7）
0	99（70.7）	累及系统数量	
1	25（17.9）	1	54（38.6）
2	12（8.6）	2	49（35.0）
3	4（2.9）	3	29（20.7）
眼睛评分		4	6（4.3）
0	83（59.3）	5	2（1.4）
1	22（15.7）	先前有无aGVHD	
2	32（22.9）	有	42（30.0）
3	3（2.1）	无	98（70.0）
胃肠道评分		是否重叠综合征	
0	127（90.7）	是	19（13.6）
1	9（6.4）	否	121（86.4）
2	4（2.9）	免疫抑制剂种类	
3	0	1	97（69.4）
肝脏评分		2	33（23.6）
0	90（64.3）	3	10（7.1）
1	27（19.3）	是否启动二线治疗	
2	20（14.3）	否	88（62.9）
3	3（2.1）	是	52（37.1）

注 NIH：美国国立卫生研究院；aGVHD：急性移植物抗宿主病

二、LSS评估的症状负荷

结果显示总体最重的症状负荷依次是眼睛［（20.48±23.75）分］、心理［（16.13±17.00）分］、口腔［（13.66±20.55）分］相关症状。7个子量表和总得分从轻度到中度、重度均依次升高，其中总分分别为（6.51±6.15）、（10.07±5.61）、（20.90±10.09）分，组间差异均有统计学意义（*F*＝32.12，*P*<0.01；采用邦弗伦尼进行事后比较，*P*值均<0.05）（图1）。

**图1:**
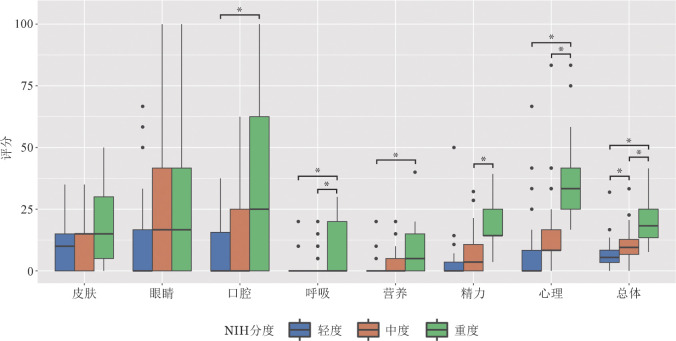
轻度、中度、重度慢性移植物抗宿主病（cGVHD）患者Lee症状学量表评估的症状负荷（*P<0.05）

三、SF-36评估的QoL

结果显示PCS得分［（40.71±7.15）分］和MCS得分［（38.35±10.19）分］均低于常模0.5个标准差以上且MCS差距更明显。所有亚项得分从轻度到重度均依次降低，且轻、重度间差异有统计学意义（图2）。多因素分析显示：总体症状负荷、口腔相关症状负荷、眼睛NIH分级、营养相关症状负荷越重，PCS越差（表2）；眼睛NIH分级越高，MCS越差（表3）。

**图2:**
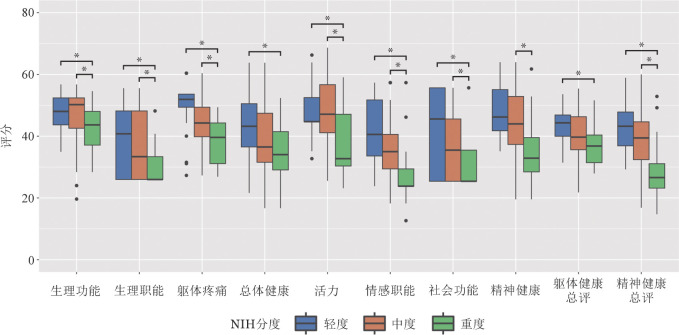
轻度、中度、重度慢性移植物抗宿主病（cGVHD）患者SF-36评估的生活质量情况（*P<0.05） SF-36：医学结局研究36项简短健康调查量表

**表2:** 影响躯体健康总评（PCS）生活质量的相关因素的线性回归分析结果

变量	单因素分析		多因素分析	
	标准化*β*（95%*CI*）	*P*值	标准化*β*（95%*CI*）	*P*值
患者相关因素				
年龄	−0.047（−0.121～0.068）	0.580		
女性	−0.098（−3.887～1.014）	0.248		
移植后评估时间（月）	−0.005（−0.067～0.063）	0.953		
发病后评估时间（月）	−0.081（−0.274～0.096）	0.344		
移植相关因素				
移植年龄（岁）	−0.049（0.068～−0.124）	0.568		
原发病（AML）				
ALL	0.006（−2.882～3.111）	0.940		
MDS	−0.139（−6.104～0.544）	0.100	−0.059（−4.358～2.005）	0.466
AA	0.069（−3.218～7.755）	0.415		
CML	0.135（−1.119～10.61）	0.112	0.143（−0.387～10.462）	0.068
其他	0.061（−3.272～7.037）	0.471		
干细胞来源（外周血 / 外周和骨髓）	0.117（−0.727～4.119）	0.169	−0.070（−3.908～1.875）	0.488
HLA匹配（全相合 / 部分相合）	0.134（−0.469～4.314）	0.114	0.103（−1.280～4.232）	0.291
性别匹配（女供男 / 非女供男）	0.016（−2.923～3.547）	0.849		
疾病相关因素				
移植后发病时间（月）	0.026（−0.062～0.085）	0.758		
整体NIH分级	0.256（−4.884～−1.086）	0.002	−0.045（−2.827～1.787）	0.656
皮肤NIH分级	0.014（−1.298～1.532）	0.871		
口腔NIH分级	−0.140（−2.842～0.252）	0.100	0.123（−1.637～3.929）	0.416
眼睛NIH分级	−0.263（−3.376～−0.796）	0.002	−0.376（−4.543～−1.425）	<0.001
胃肠道NIH分级	0.086（−1.432～4.469）	0.311		
肝脏NIH分级	0.092（−0.664～2.269）	0.281		
肺NIH分级	−0.146（−4.566～0.303）	0.086	−0.044（−4.011～2.726）	0.706
关节/筋膜NIH分级	−0.284（−6.219～−1.711）	0.001	−0.220（−6.638～0.487）	0.090
累及系统数量	−0.260（−3.189～−0.735）	0.002	−0.017（−1.639～1.384）	0.867
皮肤症状评分	−0.006（−0.105～0.098）	0.940		
眼睛症状评分	−0.048（−0.065～0.036）	0.571		
口腔症状评分	−0.188（−0.123～−0.008）	0.026	−0.456（−0.275～−0.042）	0.008
呼吸症状评分	−0.157（−0.354～0.01）	0.064	−0.135（−0.455～0.159）	0.342
营养症状评分	−0.227（−0.476～−0.077）	0.007	−0.211（−0.476～−0.038）	0.022
精力症状评分	−0.274（−0.310～−0.080）	0.001	−0.216（−0.402～0.095）	0.223
心理症状评分	−0.097（−0.111～0.030）	0.255		
总体症状评分	−0.214（−0.342～−0.045）	0.011	−0.517（0.159～0.775）	0.003
aGVHD病史（有 / 无）	0.073（−1.473～3.745）	0.391		
疾病类型（重叠综合征 / 非重叠综合征）	0.104（−1.325～5.637）	0.223		
治疗（启动二线 / 未启动二线）	−0.111（−4.103～0.828）	0.191	−0.085（−3.547～1.055）	0.286
免疫抑制剂种类	0.015（−1.770～2.128）	0.856		

注 AML：急性髓系白血病；ALL：急性淋巴细胞白血病；MDS：骨髓增生异常综合征；AA：再生障碍性贫血；CML：慢性髓性白血病；NIH：美国国立卫生研究院；aGVHD：急性移植物抗宿主病

**表3:** 影响精神健康总评（MCS）生活质量的相关因素的线性回归分析结果

变量	单因素分析		多因素分析	
	标准化*β*（95%*CI*）	*P*值	标准化*β*（95%*CI*）	*P*值
患者相关因素				
年龄	−0.067（−0.189～0.081）	0.428		
女性	0.015（−3.191～3.834）	0.857		
移植后评估时间（月）	−0.066（−0.128～0.056）	0.439		
发病后评估时间（月）	−0.178（−0.540～−0.019）	0.036	−0.116（−0.457～0.092）	0.190
移植相关因素				
移植年龄（岁）	−0.062（−0.188～0.087）	0.468		
原发病（AML）				
ALL	0.059（−2.775～5.759）	0.491		
MDS	−0.170（−9.560～−0.123）	0.044	−0.125（−8.109～0.982）	0.123
AA	0.098（−3.244～12.37）	0.250		
CML	0.017（−7.582～9.299）	0.841		
其他	−0.077（−10.715～3.973）	0.366		
干细胞来源（外周血 / 外周和骨髓）	0.007（−3.327～3.633）	0.931		
HLA匹配（全相合 / 部分相合）	0.090（−1.596～5.260）	0.293		
性别匹配（女供男 / 非女供男）	0.147（−0.538～8.591）	0.083	0.099（−1.697～7.131）	0.225
疾病相关因素				
移植后发病时间（月）	−0.004（−0.108～0.102）	0.958		
整体NIH分级	−0.399（−9.210～−4.071）	<0.001	−0.077（−4.971～2.398）	0.491
皮肤NIH分级	−0.110（−3.338～0.706）	0.200	−0.179（−5.795～1.492）	0.245
口腔NIH分级	−0.194（−4.752～−0.379）	0.022	0.092（−2.978～5.464）	0.561
眼睛NIH分级	−0.283（−5.032～−1.374）	0.001	−0.260（−5.753～−0.154）	0.039
胃肠道NIH分级	0.141（−0.633～7.731）	0.096	0.088（−2.624～7.028）	0.368
肝脏NIH分级	0.088（−0.994～3.191）	0.301		
肺NIH分级	−0.250（−8.618～−1.822）	0.003	−0.107（−7.273～2.834）	0.386
关节/筋膜NIH分级	−0.143（−6.175～0.462）	0.091	−0.032（−5.701～4.416）	0.802
累及系统数量	−0.207（−3.997～−0.451）	0.014	0.189（−0.334～4.401）	0.092
皮肤症状评分	−0.089（−0.220～0.068）	0.298		
眼睛症状评分	−0.222（−0.166～−0.025）	0.008	−0.124（−0.291～0.185）	0.660
口腔症状评分	−0.257（−0.208～−0.047）	0.002	−0.287（−0.432～0.143）	0.322
呼吸症状评分	−0.331（−0.765～−0.269）	<0.001	−0.186（−0.789～0.208）	0.251
营养症状评分	−0.216（−0.659～−0.089）	0.011	−0.140（−0.668～0.179）	0.255
精力症状评分	−0.363（−0.527～−0.209）	<0.001	−0.187（−0.621～0.243）	0.388
心理症状评分	−0.355（−0.307～−0.119）	<0.001	−0.183（−0.389～0.170）	0.438
总体症状评分	−0.447（−0.770～−0.382）	<0.001	0.339（−1.240～2.114）	0.607
aGVHD病史（有 / 无）	0.115（−1.156～6.256）	0.176	0.036（−3.143～4.748）	0.688
疾病类型（重叠综合征 / 非重叠综合征）	0.163（−0.085～9.766）	0.054	0.171（−0.465～11.126）	0.071
治疗（启动二线 / 未启动二线）	0.054（−2.399～4.668）	0.527		
免疫抑制剂种类	−0.097（−4.362～1.173）	0.257		

注 AML：急性髓系白血病；ALL：急性淋巴细胞白血病；MDS：骨髓增生异常综合征；AA：再生障碍性贫血；CML：慢性髓性白血病；NIH：美国国立卫生研究院；aGVHD：急性移植物抗宿主病

四、EQ-5D-5L评估的QoL

结果显示总体效用指数值为0.764±0.173，低于我国一般人口常模（1）。轻度（0.762±0.179）、中度（0.702±0.198）、重度（0.764±0.173）依次降低（*F*＝2.544，*P*＝0.082）。多因素分析显示，口腔相关症状负荷越重、关节/筋膜NIH分级越高、累及系统数量越多、整体NIH分级越高、服用免疫抑制剂种类越多，EQ-5D-5L评估的生活质量越差（表4）（均*P*<0.05）。进一步分析发现，EQ-5D效用指数值与SF-36量表呈中到高度相关（|*r*|＝0.384～0.571，*P*<0.001）且与PCS相关性最高（表5）。

**表4:** 影响EQ-5D-5L评估的生活质量的相关因素的线性回归分析结果

变量	单因素分析		多因素分析	
	标准化*β*（95%*CI*）	*P*值	标准化*β*（95%*CI*）	*P*值
患者相关因素				
年龄	0.016（−0.002～0.003）	0.855		
女性	−0.075（−0.086～0.033）	0.381		
移植后评估时间（月）	−0.002（−0.002～0.002）	0.980		
发病后评估时间（月）	−0.184（−0.009～0.000）	0.030	−0.083（−0.006～0.002）	0.255
移植相关因素				
移植年龄（岁）	0.015（−0.002～0.003）	0.857		
原发病（AML）				
ALL	0.036（−0.057～0.088）	0.669		
MDS	−0.095（−0.126～0.035）	0.266		
AA	0.072（−0.076～0.189）	0.398		
CML	−0.002（−0.145～0.141）	0.982		
其他	0.080（−0.065～0.183）	0.350		
干细胞来源（外周血 / 外周和骨髓）	0.081（−0.03～0.087）	0.340		
HLA匹配（全相合 / 部分相合）	0.027（−0.049～0.068）	0.754		
性别匹配（女供男 / 非女供男）	0.006（−0.075～0.081）	0.945		
疾病相关因素				
移植后发病时间（月）	0.071（−0.001～0.003）	0.407		
整体NIH分级	−0.189（−0.100～−0.007）	0.026	0.205（0.005～0.111）	0.032
皮肤NIH分级	−0.058（−0.046～0.023）	0.502		
口腔NIH分级	−0.212（−0.084～−0.011）	0.012	0.106（−0.039～0.087）	0.458
眼睛NIH分级	−0.128（−0.057～0.008）	0.133	−0.162（−0.065～0.003）	0.076
胃肠道NIH分级	0.054（−0.049～0.094）	0.527		
肝脏NIH分级	0.121（−0.010～0.061）	0.156	0.123（−0.009～0.061）	0.147
肺NIH分级	−0.254（−0.147～−0.032）	0.002	−0.121（−0.121～0.036）	0.283
关节/筋膜NIH分级	−0.417（−0.193～−0.089）	<0.001	−0.332（−0.192～−0.032）	0.006
累及系统数量	−0.440（−0.108～−0.053）	<0.001	−0.253（−0.081～−0.011）	0.010
皮肤症状评分	−0.097（−0.004～0.001）	0.257		
眼睛症状评分	−0.047（−0.002～0.001）	0.582		
口腔症状评分	−0.287（−0.004～−0.001）	0.001	−0.400（−0.006～−0.001）	0.018
呼吸症状评分	−0.255（−0.011～−0.002）	0.002	−0.081（−0.009～0.005）	0.534
营养症状评分	−0.250（−0.012～−0.003）	0.003	−0.088（−0.007～0.002）	0.283
精力症状评分	−0.446（−0.010～−0.005）	<0.001	−0.183（−0.008～0.002）	0.241
心理症状评分	−0.239（−0.004～−0.001）	0.004	−0.136（−0.004～0.001）	0.317
总体症状评分	−0.359（−0.011～−0.004）	<0.001	0.316（−0.003～0.016）	0.156
aGVHD病史（有 / 无）	0.077（−0.034～0.092）	0.367		
疾病类型（重叠综合征 / 非重叠综合征）	0.067（−0.051～0.118）	0.433		
治疗（启动二线 / 未启动二线）	0.012（−0.056～0.064）	0.886		
免疫抑制剂种类	−0.114（−0.079～0.015）	0.178	−0.171（−0.088～−0.008）	0.019

注 EQ-5D-5L：五水平五维健康量表；AML：急性髓系白血病；ALL：急性淋巴细胞白血病；MDS：骨髓增生异常综合征；AA：再生障碍性贫血；CML：慢性髓性白血病；NIH：美国国立卫生研究院；aGVHD：急性移植物抗宿主病

**表5:** EQ-5D效用指数值与SF-36亚项间的相关性分析

SF-36亚项	*r*值	*P*值
生理功能	0.495	<0.001
生理职能	0.416	<0.001
躯体疼痛	0.518	<0.001
总体健康	0.413	<0.001
活力	0.495	<0.001
社会功能	0.384	<0.001
情感职能	0.389	<0.001
精神健康	0.393	<0.001
躯体健康总评	0.571	<0.001
精神健康总评	0.428	<0.001

注 EQ-5D：五水平五维健康量表；SF-36：医学结局研究36项简短健康调查量表

## 讨论

cGVHD发生机制复杂，临床表现多样，病程迁延持久，严重影响患者的QoL和远期生存。随着移植技术的发展及cGVHD防治手段的完善，患者对移植后QoL的诉求越来越高，了解并干预QoL是移植后重要的管理方向。

本研究我们发现，LSS总评分在不同严重程度患者间差异具有统计学意义，而具体到皮肤、眼睛症状差异无统计学意义；SF-36的所有量表在轻、重度cGVHD间差异有统计学意义且MCS在重度与轻、中度间差异有统计学意义，PCS与EQ-5D效用指数值具有较高的相关性。根据本研究结果我们认为，患者的症状负荷可采用LSS进行初筛，具体症状需要专科量表优化；重度患者的精神QoL可采用MCS识别，躯体、精神QoL的精细评估仍待优化。

与国外研究不同，总体患者的症状负担主要为眼睛、心理和口腔且以MCS受损为著；而美国某队列研究的症状负担主要为皮肤、精力和心理[22]，另有队列为眼睛、精力和心理[23]，可见不同国度间患者存在差异；有研究表明躯体较心理QoL受损更为严重[12]，我国尚无类似研究报道。以上不同队列的差异提醒我们在相同NIH分级下采用PRO的必要性，从而评估超出临床医师对于严重程度认识的广泛问题。我们的数据显示总体症状负荷、眼睛受累程度分别显著影响躯体、心理QoL；而有研究发现肺、胃肠等轻度症状与躯体QoL恶化有关[13]，说明在cGVHD发病的基础上应更加关注累及部位带来的影响，强调靶向器官精准治疗的必要性。本研究结果显示口腔相关症状负荷、关节/筋膜严重程度等显著影响健康效应指数，提示在临床中注意改善口腔症状、康复关节护理等可以从一定程度上改善患者的健康状态，建议加强亚学科合作和非药物干预的管理。

总之，这项研究首次展示我国cGVHD患者QoL的详细数据，推荐LSS作为症状负担评估工具，具体症状及具体领域的生活质量评估仍亟待优化，特别呼吁建立中国患者的评估量表和计算常模，继本研究后我们将纳入多中心患者入组验证，进而推进cGVHD患者QoL管理的临床实践。
